# FROM REACH TO MINDFULNESS: SHIFTING PARADIGMS IN SUPPORT OF CAREGIVERS OF VETERANS LIVING WITH DEMENTIA

**DOI:** 10.1093/geroni/igae098.1779

**Published:** 2024-12-31

**Authors:** Jyoti Savla, Mamta Sapra, Katherine Luci, Lauren Hagemann

**Affiliations:** Virginia Tech, Blacksburg, Virginia, United States; Salem VA Healthcare System, Salem, Virginia, United States; Salem VA Healthcare System, Salem, Virginia, United States; Salem VA Healthcare System, Salem, Virginia, United States

## Abstract

Enhancing interventions to alleviate stress among caregivers is a key area of focus for the Veterans Health Administration, especially given the anticipated tripling of dementia cases among Veterans in the upcoming years. Existing programs, like REACH-VA, have focused on cognitive behavioral approaches to reduce caregiver strain. However, mindfulness practices, known for stress reduction, psychological well-being improvement, and relationship enhancement, have not been extensively explored for caregivers of Veterans. This study evaluated the comparative effectiveness of a four-session, multi-faceted mindfulness-based intervention called Practice of Acceptance, Awareness, and Compassion in Caregiving (PAACC) to REACH-VA program for family caregivers of Veterans living with dementia. PAACC combines mindfulness with caregiver skill-development to improve the caregiving experience. It aims to assist caregivers in developing immediate awareness of stressors and their responses to stressors, while fostering empathy towards their relative with dementia. This approach differs from strategies that challenge or replace negative or unrealistic stress-related thoughts, considering the often progressive and unchangeable nature of such stressors. A randomized-controlled trial involving 133 family caregivers (Mage = 67 years; 81% White; 89% co-residing) assigned to either REACH-VA or PAACC demonstrated significant reductions in perceived stress, caregiver burden, and rumination after participating in either of the interventions. However, multi-level models revealed that caregivers who received PAACC showed lower anxiety (B=-2.76, p<0.001) and depressive symptoms (B=-2.50, p<0.01), and higher mindfulness (B=6.04, p<0.01) compared to caregivers who received the REACH-VA intervention. Findings will be discussed in the context of the intervention’s mechanisms, lessons learned, and alternative avenues for utilizing PAACC.

